# Factors affecting the outcome of human blastocyst vitrification

**DOI:** 10.1186/1477-7827-7-99

**Published:** 2009-09-16

**Authors:** Amr A Kader, Audrey Choi, Yasser Orief, Ashok Agarwal

**Affiliations:** 1 Center for Reproductive Medicine, Glickman Urological and Kidney Institute, Ob/Gyn and Women's Health Institute, Cleveland Clinic, Cleveland, Ohio, USA; 2 Department of Obstetrics and Gynecology, University of Alexandria, Alexandria, Egypt; 3 Center of Surgical Innovation, Technology and Education, Cleveland Clinic, Cleveland, Ohio, USA; 4 Case Western Reserve University School of Medicine, Cleveland, Ohio, USA

## Abstract

With single blastocyst transfer practice becoming more common in ART, there is a greater demand for a convenient and reliable cryostorage of surplus blastocysts. Vitrification has emerged in the last decade as an alternative promising substitute for slow freezing. Blastocysts represent a unique challenge in cryostorage due to their size, multicellular structure and presence of blastocoele. The continuous acquisition of experience and introduction of many different technological developments has led to the improvement of vitrification as a technology and improved the results of its application in blastocyst cryostorage. The current information concerning safety and efficacy of the vitrification of blastocysts will be reviewed along with the variables that can impact the outcome of the procedure.

## Background

With the refinement of extended culture systems, it is becoming more reliable to obtain blastocysts in vitro [1]. Due their high implantation rates, it is becoming a common practice to limit transfer to one or two blastocysts at a time. Therefore, surplus blastocysts require an efficient cryopreservation method [2,3]. Slow freezing was the main method of cryopreservation [4], but vitrification is now on the rise. Vitrification is the glass-like solidification of a solution at a low temperature without ice crystal formation, which is made possible by extreme elevation in viscosity during freezing. This can be achieved by increasing the freezing and warming rates and/or increasing the concentration of the cryoprotectants [5]. Unlike slow freezing, vitrification results in the total elimination of ice crystal formation, both within the cells being vitrified and outside the cells in the surrounding solution [6]. Although high concentrations of cryoprotectants can be toxic, and the vitrified solution is prone to glass fractures, these effects can be controlled by adjusting the vitrification protocol and technique. With vitrification, the blastocyst is combined with cryoprotectants that maximize cytoplasmic viscosity while exerting a strong dehydrating effect. Vitrification is more convenient and is possibly superior because it avoids ice crystal formation. Over the last decade, vitrification techniques have been standardized, tested and improved via controlled experiments designed to elucidate the optimal conditions under which vitrification should be performed. This review will discuss the most commonly used loading devices, vitrification safety in terms of perinatal outcomes, and the factors that can affect the success of human blastocyst vitrification.

## Human blastocysts vitrified using different loading devices

During vitrification, the blastocyst is placed in a loading device surrounded by vitrification media. The device is then placed into liquid nitrogen, where it is stored. There are a variety of loading devices available today: the Cryoloop, Cryotop, Cryoptip, Cut Standard Straws, Cryo-leaf™ and High Security Straws™. The Cryoloop is a nylon loop, whereas the Cryotop is a plastic container. These are considered open systems because the blastocysts come into direct contact with the liquid nitrogen. Cryotips are plastic straws with protective metal sleeves and is heat sealed from both ends after loading, thus constituting a closed system. The cut standard straw is a system that can be used as an open method (by direct contact with liquid nitrogen) or closed if placed inside a sealed standard straw (straw within straw). The Cryo-leaf™ is a plastic carrier open system, vitrifying the specimen by direct contact. High security straws are plastic straws sealed after loading, and are thus considered a closed system. Table 1 summarizes the survival, implantation and pregnancy rates of human blastocysts vitrified using different loading devices.

**Table 1:** Comparison of survival, implantation and pregnancy rates according to loading device

	**Loading Device**	**Sample Size**	**Survival Rate**	**Implantation Rate**	**Pregnancy Rate**
**Mukaida *et al*, 2001**[8]	Cryoloop	N = 60	63%	--	31%
**Cho, 2002 *et al ***[21]	EM grid	N = 21	83%	--	34%
**Reed *et al*, 2002**[10]	Cryoloop	N = 54	100%	15%	--
**Mukaida *et al*, 2003**[9]	Cryoloop	N = 725	80%	20%	37%
**Osada *et al*, 2003**[11]	Cryotop	N = 580	99%	--	56%
**Stehlik *et al*, 2005**[12]	Cryotop	N = 41	100%	--	50%
**Takahashi *et al*, 2005**[19]	Cryoloop	N = 1129	86%	29%	44%
**Kuwayama *et al*, 2005**[18]	Cryotip	N = 5695	90%	--	53%
**Liebermann *et al*, 2006**[13]	Cryotop	N = 547	97%	31%	46%
**Mukaida *et al*, 2008**[29]	Cryoloop	N = 5412	92%	36%	49%

In 1999, Lane *et al *[7] reported that human blastocysts vitrified by cryoloop had hatching rates similar to those of fresh blastocysts. Mukaida *et al *[8,9] and Reed *et al *[10] vitrified blastocysts using the Cryoloop, producing survival rates ranging from 63% to 100% and pregnancy rates ranging from 31% to 37%. In 2001, Mukaida *et al *reported the first successful delivery of three healthy newborns who had been conceived via blastocyst vitrification using the Cryoloop [8].

In 2003, Osada *et al *[11] studied the vitrification of blastocysts using the Cryotop™ and reported 99% survival rate and 56% pregnancy rate, which was even higher than the 31% pregnancy rate in their fresh blastocyst transfer group. Stehlik *et al *[12] and Liebermann and Tucker [13] compared vitrification by Cryotop™ with conventional slow freezing methods. Liebermann and Tucker [13] did not find a statistically significant difference in survival and pregnancy rates between blastocysts vitrified by the Cryotop™ and those cryopreserved by slow freezing. On the other hand, Stehlik *et al *[12] reported that survival and pregnancy rates of blastocysts vitrified by the Cryotop™ significantly exceeded the rates of blastocyst survival after slow cryopreservation.

Despite the wide use and successful vitrification of human and animal oocytes and embryos using open pulled straws (OPS) [14,15], only modified OPS were used by Cremades et al [16] and resulted in survival rate of 82% in a small sample of 33 human blastocysts.

In 2005, Kuwayama *et al *[17] performed a study that validated the use of the Cryotip™ for the first time, reporting that the Cryotip™ produced results that were comparable to those of the Cryotop™ carrier. The Cyrotip™ demonstrated 93% blastocyst survival rate and 51% pregnancy rate with no statistical difference when compared with the rates of the Cryotop™ [18].

In 2005, Takahashi *et al *[19] reported the clinical outcomes of a 4-year study on 1129 vitrified human blastocysts using the cryoloop. This large sample size demonstrated that the pregnancy rate and implantation rates using vitrified blastocysts were comparable to those associated with use of fresh blastocysts.

In a recent report by Liebermann et al [20], of 8,449 blastocysts from 2,453 patients that were vitrified, 1398 vitrified blastocysts were transferred with a survival rate of 96.3%, an implantation rate of 29.4%, and a clinical pregnancy rate per frozen embryo transfer of 42.9%.

Blastocysts can also be vitrified on an electronic microscope (EM) copper grid. Cho *et al *[21] reported vitrifying human blastocysts in this manner with a survival rate of 83% and a pregnancy rate of 34%.

## Obstetric and perinatal outcomes

Multiple pregnancy is the main source of obstetric and perinatal morbidity associated with assisted reproduction. The transfer of blastocysts allowed one or two blastocysts to be transferred with high implantation potential, while minimizing the risks of multiple pregnancies. Single blastocyst transfer completely avoids dizigotic twin pregnancy [1,22-25].

Vitrification has been in clinical use for more than 15 years. And while multiple studies have reported excellent cryosurvival and pregnancy rates using vitrified oocytes or embryos, there are still concerns regarding the overall safety of vitrification and whether it can cause or lead to chromosomal abnormalities, congenital malformation, and/or developmental abnormalities in the offspring [26,27]. As a result, no general recommendation in favor of its regular clinical use has been issued.

Part of the problem is a lack of well-controlled clinical trials. Noyes et al [28] reviewed a total of 58 reports (1986-2008) on 900 cryopreserved oocytes looking for data on congenital anomalies in 609 live born babies (308 from slow-freezing, 289 from vitrification and 12 from both methods). Twelve newborns (1.3%) had birth anomalies, which is comparable to the number of congenital anomalies that occur in naturally conceived infants. Analyzing the obstetric and perinatal outcomes following transfer of vitrified blastocysts would be even more challenging due to the limited number of reports, though this number is rapidly rising.

Takahashi *et al *[19] reported congenital birth defects of 1.4% using vitrified blastocysts which was similar to fresh blastocysts. In a preliminary report on the effect of blastocyst vitrification on perinatal outcomes, Mukaida *et al *[29] analyzed 560 deliveries of 691 healthy babies following the transfer of vitrified blastocysts. The congenital and neonatal complication rate was 3%, which was comparable to that in their fresh blastocysts transfer group (2.3%). No perinatal abnormalities were reported in Liebermann's report on 348 deliveries of 431 babies following transfer of vitrified blastocysts [20].

These findings may provide preliminary reassurance on the safety of blastocyst vitrification. A final verdict on the actual effect of blastocyst vitrification on congenital and perinatal outcomes may not be possible until large-scale trials or further meta-analysis of rapidly accumulating reports can be performed.

## Factors that can affect the outcome

There are a number of variables that can determine the outcomes of vitrification:

•Pre-vitrification blastocyst selection

•Post-thaw blastocyst selection

**Figure 1:**
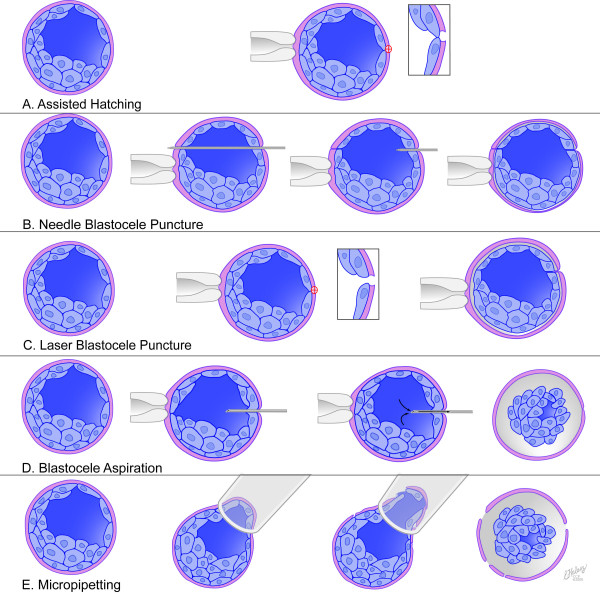
**Different pre-vitrification interventions for blastocysts**. A. Assisted hatching: An opening is created in the zona using laser pulse B. Needle blastocoele puncture: A needle is passed through the zona and blastocoele and retracted allowing the blastocelic fluid to freely leak. C. Laser blastocoele puncture: laser pulse creates an opening in the zona and a small defect in the trophectoderm causing the blastocoele to leak. D. Blastocoele aspiration: An injection needle is introduced into the blastocoele and blastocoelic volume is sucked out. E. Micropipetting: Passing the blastocysts through a narrow pipette would crack the zona and compress the blastocoele to leak through the cracked zona.

•Assisted hatching (Figure 1)

•Blastocoele collapse (assisted shrinkage) (Figure 1)

•Media protocols

•Freezing rate

•Warming rate

•Operator-dependent factors

•Hydrostatic pressure

## Pre-vitrification blastocyst selection

Selection focuses on the quality of the original embryo and/or the time at which the blastocyst is vitrified.

### Influence of early embryonic quality

The quality of an early embryo determines the quality of the blastocyst, and therefore the outcome of the blastocyst vitrification. In a study by Vanderzwalmen *et al *[30], vitrified blastocysts that originated from a cohort of early embryos with less than 30% fragmentation had survival, implantation and ongoing pregnancy rates of 73%, 32% and 19%, respectively. In contrast, when the blastocysts came from embryos with 30-50% fragmentation and/or unequally sized blastomeres, these rates decreased to 38%, 9% and 6%, respectively. These findings highlight the importance of following the day-by-day development of each embryo so that the outcome of blastocyst vitrification and later transfer can be predicted.

### Day 5 versus day 6 vitrification

Blastulation of human embryos usually occurs on day 5 after fertilization but may be delayed until day 6. The transfer of *fresh *day-5 blastocysts seems to result in higher pregnancy rates than the transfer of fresh day-6 blastocysts [11,31,32]. However, the transfer of slowly *cryopreserved *day-6 blastocysts results in comparable pregnancy rates to the transfer of cryopreserved day-5 blastocysts [13,33]. This may be related to better endometrial synchrony in the cryopreserved blastocyst transfer cycles; the endometrial receptivity window may be missed in day 6 fresh transfer [34].

Table 2 summarizes the different studies that have compared day-5 with day-6 blastocyst cryopreservation.

**Table 2:** Different studies comparing the slow preservation and/or vitrification of day 5 and day 6 blastocysts in terms of survival after warming, implantation and pregnancy rates

	**Slowly frozen Day 5 Blastocysts**	**Slowly frozen Day 6 Blastocysts**	**Vitrified Day 5 Blastocysts**	**Vitrified Day 6 Blastocysts**
**Mukaida *et al. *2003**[9]			Survival 87%	Survival 55%
**Stehlik *et al. *2005**[12]	Survival 83.1% Pregnancy rate 16.7%	Survival 89.5% Pregnancy rate 18.5%	Survival 100% Pregnancy rate 50%	Survival 100% Pregnancy rate 33%
**Liebermann & Tucker 2006**[13]	Survival 91.4% Implantation 29.6% Pregnancy rate 42.8%	Survival 94.8% Implantation 28.2% Pregnancy rate 43.1%	Survival 95.9% Implantation 33.4% Pregnancy 48.7%	Survival 97.5% Implantation 25.9% Pregnancy 42.8%
**Kader *et al. *2008**[36]	DNA integrity index: 94.76% ± 4.70	DNA integrity index: 90.87% ± 6.16	DNA integrity index: 84.36% ± 8.76	DNA integrity index: 77.61% ± 16.65

In different clinical studies, day-5 blastocysts were generally associated with better outcomes following cryopreservation by vitrification than day-6 blastocysts. Mukaida *et al *and Veeck *et al *reported superior survival rates with blastocysts vitrified on day 5 compared with those vitrified on day 6 [9,35]. In a study with 41 vitrified blastocysts, Stehlik *et al *[12] reported a pregnancy rate of 50% using vitrified day-5 blastocysts, compared with a 33% pregnancy rate using day-6 blastocysts. Liebermann and Tucker [13] found that implantation and pregnancy rates were significantly higher after the transfer of day-5 vitrified blastocysts than after transfer with day-6 blastocysts. However, they did not find a statistically significant difference in survival rates between the two groups. The results of slow cryopreservation of day-5 versus day-6 blastocysts were similar, although no statistical significance between the two groups was reached.

We have recently shown that day-5 blastocysts have less DNA damage than day-6 blastocysts, although the difference was not statistically significant due to a limited sample size [36].

The superior outcomes associated with vitrified day-5 blastocysts may be related to the fact that many of the day-6 blastocysts were delayed in development, suggesting that they were of inferior quality. In the case of expanded good quality day 6 blastocysts, damage could still be explained by an increase in number of blastomeres, increase in their metabolic activity and an increase in blastocoele expansion. Any of these factors could increase the likelihood of inadequate vitrification, ice crystal formation, and cryodamage [36,37]. Therefore, embryos that undergo blastulation on day 5 would better be vitrified on day 5, while embryos delayed in development may be allowed to develop to day 6 until vitrified. The rate of development and the degree of expansion are more likely to affect the outcome than the day of vitrification [13,36]. After all, transferred vitrified embryos will benefit from a better endometrial synchrony, which may dampen negative effects from cryostorage [34].

## Post-thaw blastocyst selection

Post-warming, viable blastocysts re-expand and are usually allowed four to six hours of incubation to regain their vitality before being transferred. Re-expansion is the sign of viability. An important predictor of the transfer of vitrified-warmed blastocyst is the blastocyst re-expansion timing. The earlier the blastocyst expands, the better it is expected to perform after transfer [38].

## Assisted hatching

Pribenszky *et al *[39] studied the survival of zona-free mouse blastocysts. There was no difference in survival after thawing between these blastocysts and fresh control blastocysts. This experiment suggested that the intact zona pellucida can potentially negatively impact blastocyst vitrification

In lieu of using zona-free blastocysts, which may not be practical with human blastocysts, assisted hatching can be performed prior to vitrification. With assisted hatching, a small hole is created in the zona pellucida so that the blastocyst can more easily escape or "hatch." It was primarily thought to overcome the post-freezing zonal hardening preventing spontaneous hatching and it proved effective [30].

Assisted hatching has been shown to improve the outcome of vitrification of blastocysts through another mechanism. Applying assisted hatching prior to blastocyst vitrification allows better permeation of the cryoprotectants and better blastocoele dehydration [36,40]. Zech *et al *[40] found that vitrified warmed blastocysts that had undergone assisted hatching had significantly better survival, implantation and pregnancy rates than blastocysts with an intact zona. In concordance with Zech's findings, we have demonstrated that assisted or spontaneous hatching both have a significantly positive impact on the post-warming DNA integrity index of mice blastocysts post-warming as compared with zona-intact blastocysts [36]. These two studies show that assisted hatching is a useful and effective pre-vitrification intervention that can reduce DNA damage incurred during the vitrification process and improve clinical outcome parameters.

Table 3 summarizes the results of studies assessing the outcomes of pre-vitrification assisted hatching.

**Table 3:** Studies showing different methods of blastocyst pre-vitrification interventions and their outcome parameters

**Blastocoele Evacuation**						
**Authors, year**	**Species**	**Method**	**Intervention Sample size**	**Outcome parameter**	**Intervention**	**Control**
**Vanderzwalmen *et al. *2002**[37]	Human	Micro-needle puncture	N = 75	Survival rate	70.6%	20.3%
				Pregnancy rate	20.5%	4.5%
				Implantation rate	18.4%	7.1%
**Son *et al. *2003**[43]	Human	Micro-needle puncture	N = 90	Survival rate	90.0%	---
				Pregnancy rate	48.0%	---
				Implantation rate	29.0%	---
**Hiraoka *et al. *2004**[44]	Human	Micropipetting	N = 48	Survival rate	98.0%	---
				Pregnancy rate	50.0%	---
				Implantation rate	33.0%	---
**Chen *et al. *2005**[42]	Mice	Microsuction	N = 108	Survival rate	92.0%	80.0%
**Mukaida *et al. *2006**[41]	Human	Microneedle puncture	N = 462	Survival rate	97.2%	85.0%
				Pregnancy rate	60.2%	34.1%
				Implantation rate	46.5%	---
**Mukaida *et al. *2006**[41]	Human	Laser pulse	N = 40	Survival rate	97.5%	85.0%
				Pregnancy rate	61.5%	34.1%
				Implantation rate	48.6%	---
**Kader *et al. *2009**[45]	Mice	Microsuction	N = 22	DNA integrity index	90.1%	77.6%
**Zonal Hatching**						
**Author, year**	**Species**	**Method**	**Intervention Sample size**	**Outcome parameter**	**Intervention**	**Control**
**Zech *et al. *2005**[40]	Human	Spontaneous and Assisted (Mechanically)	N = 38	Survival rate	82%	64%
				Pregnancy rate	35%	21%
				Implantation rate	26%	12%
**Kader *et al. *2009**[45]	Mice	Assisted (Acidified Tyrod's)	N = 16	DNA integrity index	94.6%	84.4%
		Spontaneous	N = 12	DNA integrity index	88.5%	77.6%

## Blastocoele collapse (assisted shrinkage)

Much attention has been paid to the volume of the blastocoele prior to vitrification and its effect on the overall success of vitrification. A negative correlation between blastocelic volume and outcome measures has been attributed to an increased likelihood of intracellular ice formation in an inadequately dehydrated blastocoele [41,42]. Consequently, a process called assisted shrinkage was developed to reduce blastocelic volume prior to vitrification. Assisted shrinkage can be performed in a variety of ways, including micro-needle puncture of the zona pellucida [37,41,43], laser-pulse opening of the zona pellucida [41], repeated micropipetting of the blastocoele [44], and microsuction of the blastocoelic contents [42,45]

Mukaida *et al *[41] reported significant improvements in clinical outcome measures in blastocysts that had undergone assisted shrinkage as compared with a retrospective vitrification control group. There were no statistical differences in survival, implantation and clinical pregnancy rates between blastocysts that had undergone laser pulse opening or micro-needle puncture [41]. Vanderzwalmen *et al *and Son *et al *have also reported improved results using micro-needle puncture of blastocysts prior to vitrification [37,43].

Hiraoka *et al*, [44] mechanically collapsed blastocysts by repeated micropipetting prior to vitrification. The investigators reported 98% survival rate, 33% implantation rate, and 50% pregnancy rate in a sample of 48 vitrified blastocysts.

Chen *et al *[42] reported significant improvement in survival rates in blastocysts treated with blastocoelic microsuction prior to vitrification. The non-expanded blastocyst survival rate improved significantly with microsuction, and the survival rate for the expanded blastocysts improved from 59% to 89%. We have previously demonstrated significant improvement in the DNA integrity index by microsuction of mice blastocysts prior to vitrification compared with blastocyst vitrification without any pre-intervention [45]

Table 3 summarizes the results of studies assessing the outcomes of pre-vitrification assisted shrinkage.

## Improvement in media protocols

Since the inception of vitrification as a technique, many different media protocols have been tested to achieve proper intracellular cryoprotectant delivery.

### Single versus multiple cryoprotectants

In the early 1990s, investigators often used single exposure to a highly concentrated solution composed of one cryoprotectant. In 1991, Li and Trounson [46] found that the use of dimethyl sulfoxide (DMSO), 1,2-propanediol and glycerol in combination yielded better post-thaw blastocyst survival rate (61%) than when either cryoprotectant was used alone. With two cryoprotectants, the concentration of each can be lower than that needed when either is used separately, thereby making the solution less toxic to the blastocysts.

### Macromolecules

Extracellular disaccharides and macromolecules, such as sucrose and Ficoll are commonly added to vitrification solutions. This helps draw water out of the blastocoele to attain better dehydration and reduce osmotic shock. The addition of macromolecules also means that the concentration of cryoprotectants can be lowered [14,47].

### Single versus multiple steps

A single exposure to a cryoprotectant subjects the blastocyst to an increased risk of osmotic shock, particularly when the concentration is extremely high. Depending on the duration of exposure, a single immersion may not allow enough time for adequate cryoprotectant permeation into the blastocoele. Survival rates after vitrification improved with the evolution of two-step protocols. In the two-step protocols, the blastocyst is allowed to equilibrate for a few minutes at a lower cryoprotectant concentration before a short exposure to the vitrification solution at a higher concentration [14]. This enables the cryoprotectants to more gradually and effectively permeate the blastocysts while reducing the risk of osmotic shock and toxicity. Investigators comparing one-step and two-step protocols demonstrated significantly improved survival rates ranging from 70% to 90% with the two-step method [48-50].

Survival and hatching rates tend to decline when the concentrations of cryoprotectants become too high, especially in the blastocyst stage, which requires a delicate balance between high cryoprotectant delivery and ensuing cellular toxicity. One of the most commonly used protocols consists of an equilibrium solution of 7.5% ethylene glycol (EG) and 7.5% DMSO mixture, followed by a vitrification solution of 15% EG and 15% DMSO [13,41,44]. Protocols that use combinations of cryoprotectants at very high concentrations tend to have lower survival and hatching rates [51,52].

### Media volume

Using a small volume of media expedites heat transfer by minimizing the freezing or warming propagation time. Theoretically, a very small drop (~ 5 nL) of pure water should vitrify, if cooled very rapidly [53]. The freezing rate is slower when larger drops are used. In the presence of impurities or a temperature above the glass transition temperature (-140°C), ice nucleation is likely to occur. Ice nucleation is a critical event and must be avoided since a single nucleation event in the liquid material before vitrification is reached will trigger crystallization of the specimen [54].

In order to achieve the maximal freezing rates, current vitrification loading devices hold a minimal volume of solution such as the EM grid, cryoloop™, cryotip™, and Cryo-leaf™ high security straws.

Currently most acceptable target in designing vitrification loading devices for oocytes or embryos is to use a small volume (<1 μl) of high-concentration cryoprotectant (~ 30%), and very rapid freezing rates of 15,000 to 30,000°C/min [55].

## Freezing rate

A high freezing rate is crucial to achieving proper vitrification and survival. This can be achieved via direct contact between the sample and liquid nitrogen or indirect contact if the sample is contained in a closed carrier.

### Direct contact vitrification

In this method, a high freezing rate is achieved by avoiding any delay that may be caused by the carrier walls. This method was considered the gold standard for vitrification until concerns about liquid nitrogen contamination led researchers to develop closed systems [56,57]. The EM grid is an example of an old open method.

### Closed system vitrification

In a closed system, the specimen is not allowed to directly come in contact with the liquid nitrogen. Therefore, a carrier is required to deliver the maximum heat transfer rate to the contained specimen. Closed containers try to achieve this minimal impedance of heat transfer by design (being ultrathin, containing microvolumes) and by material selection. The most recent developments in the closed systems are the CryoTip™ and Cryo-leaf™ the high security straws (HSS).

Cut standard straws hold blastocysts in a 0.75 μl chamber with a freezing rate of 15,000°C/min if open and 600°C/min if closed. Isachenko *et al *[58] did not report any difference in the survival rate of blastocysts vitrified in the open or closed system. This demonstrates that vitrification can occur at a lower-than-expected freezing rate.

An alternative way to increase the freezing rate is to decrease the temperature of the liquid nitrogen. This increases the freezing through two mechanisms: (1) the wider difference in temperature leads to more rapid transfer and (2) it minimizes the chances of insulating gas bubble formation. Two mechanisms have been described to decrease the nitrogen temperature:

1. Vacuum application over the liquid nitrogen would decrease the liquid nitrogen temperature to range between -200°C to -210°C as a result of elimination of heating and evaporation at the liquid/gas interface [54,59-61].

2. Nitrogen slush with a temperature of -210°C is less likely to evaporate on contact with the specimen compared to liquid nitrogen, [62].

## Warming rate

Proper warming is as important as rapid freezing to achieve proper vitrification-devitrification [54]. This is usually done with the immediate transfer of the sample to a pre-warmed (37°C) environment while making sure this temperature is immediately available to the sample. This can be done in open methods by mixing the sample in pre-warmed media or in closed methods by plunging the sample in its loading device into a warm water bath. The heating rate will be controlled by the same factors that control the freezing rate.

Because dilution of the cryoprotectants and re-expansion of the blastocoele occur during the warming process, it is necessary to perform the process using a series of media with gradually decreasing osmotic pressure in an effort to reduce osmotic shock [21]. One commonly used warming protocol uses three steps, beginning with 0.3 mol/L sucrose in base medium, followed by transfer to 0.2 mol/L sucrose in base medium, and finally to a solution containing only base medium [41].

## Operator factors

The vitrification outcome is highly operator dependent, and it requires a totally different skill set than is needed with slow freezing. The embryologist should be rapidly handling the embryos in micro-volumes of highly viscous media. Also, because there are a variety of loading devices available, specific training on the use and storage of a certain device and standardization of quality control procedures is mandatory. The embryologist should be well oriented to the different critical procedural details that can affect the vitrification outcome. Those details can be summarized as follows:

1. The types and concentrations of cryoprotectants used and their toxicity threshold

2. The temperature of the vitrification solution at exposure

3. Avoidance of media mixing in multi-step protocols

4. The duration of exposure to the final cryoprotectants before plunging into LN_2_

5. The rapid loading

6. Sealing in a closed system

7. System validation (loading, sealing, storage)

## Future perspectives

Researchers are currently studying different methods to improve vitrification outcome by manipulating the essential factors (Cryoprotectants concentrations, constituents, freezing rate, warming). The vitrification of embryos has shown to be successful at low cryoprotectant concentration and increased rate of freezing. [63].

Simultaneously, non traditional tools such as the effect of high hydrostatic pressure (HHP) in the pre-treatment of oocytes and embryos, including blastocysts to improve vitrification outcomes is also under investigation. Research has shown that HHP leads to the production of heat shock proteins in mammalian cells [64], which could potentially provide enough cellular protection to maintain homeostasis and even improve cryoprotection [65]. The types and amount of such proteins synthesized in the stressed cells depend on the intensity and type of the heat shock as well as on the stressed cell type and state.

Recent studies have reported promising results when applying HHP prior to vitrification of murine blastocysts, mature porcine oocytes and boar semen [39,66-69]. For example, applying hydrostatic pressure of 60 MegaPascals (MPa) for 30 minutes then allowing four to five minutes before vitrification significantly improved the survival and hatching rates of vitrified murine blastocysts [68].

The pressure level, pressure duration, temperature at time of pressurizing, and recovery time before vitrification are important parameters that need to be properly identified for oocytes, embryos, and blastocysts of different species [69]. However, further studies would be required to fully understand and control this phenomenon as well as to standardize its use. The use of high hydrostatic pressure before vitrification is still under investigation.

## Conclusion

Vitrification of blastocysts can be successfully carried out using many loading devices. It could eventually replace slow freezing of blastocysts as suggested by various reports in the literature[70,71] Though effect on perinatal outcome has not been fully investigated due to the novelty of the technique in clinical practice, however, the available data supports its potential safety. Other than the patient clinical parameters, the clinical success of transferring vitrified blastocysts would rely on a multitude of factors. The selection of a good quality embryo on preferably day 5 post fertilization is the 1^st ^step. The selection of blastocysts that show earlier re-expansion post-thaw for transfer could improve the outcome from transferring vitrified blastocysts. The assisted hatching and induction of blastocoele collapse prior to vitrification have also shown to improve the blastocyst vitrification outcome. Current media protocols and loading devices are capable of achieving proper vitrification attaining high level of viscosity and dehydration of the blastocysts and delivering high freezing and warming rates. Still further developments in vitrification media and devices are possible. Finally, the embryologist training would have a major bearing on the vitrification outcome.

## Abbreviations

DNA: DeoxyRibonucleic Acid; nL: nanoLiter; C: Centigrade; mol/L: mole/liter; HHP: High Hydrostatic Pressure; MPa: MegaPascal = 10 times atmospheric pressure.

## Competing interests

The authors declare that they have no competing interests.

## Authors' contributions

AK has made substantial contributions to conception and design; to the acquisition and interpretation of data; and in drafting and revising the manuscript for intellectual content. AC has made substantial contributions to the acquisition of data and drafting the manuscript. YO has made substantial contributions to the acquisition of data and drafting the manuscript. AA has made substantial contributions revising the review critically for important intellectual content; and has given final approval of the version to be published.

All authors have read and approved the manuscript.

## References

[B1] Jones GM, Trounson AO, Lolatgis N, Wood C (1998). Factors affecting the success of human blastocyst development and pregnancy following in vitro fertilization and embryo transfer. Fertil Steril.

[B2] Sunde A (2007). Significant reduction of twins with single embryo transfer in IVF. Reprod Biomed Online.

[B3] Cutting R, Morroll D, Roberts SA, Pickering S, Rutherford A (2008). Elective Single Embryo Transfer: Guidelines for Practice British Fertility Society and Association of Clinical Embryologists. Hum Fertil (Camb).

[B4] Menezo Y, Nicollet B, Herbaut N, Andre D (1992). Freezing cocultured human blastocysts. Fertil Steril.

[B5] Fahy GM, MacFarlane DR, Angell CA, Meryman HT (1984). Vitrification as an approach to cryopreservation. Cryobiology.

[B6] Rall WF (1987). Factors affecting the survival of mouse embryos cryopreserved by vitrification. Cryobiology.

[B7] Lane M, Schoolcraft WB, Gardner DK (1999). Vitrification of mouse and human blastocysts using a novel cryoloop container-less technique. Fertil Steril.

[B8] Mukaida T, Nakamura S, Tomiyama T, Wada S, Kasai M, Takahashi K (2001). Successful birth after transfer of vitrified human blastocysts with use of a cryoloop containerless technique. Fertil Steril.

[B9] Mukaida T, Nakamura S, Tomiyama T, Wada S, Oka C, Kasai M, Takahashi K (2003). Vitrification of human blastocysts using cryoloops: clinical outcome of 223 cycles. Hum Reprod.

[B10] Reed ML, Lane M, Gardner DK, Jensen NL, Thompson J (2002). Vitrification of human blastocysts using the cryoloop method: successful clinical application and birth of offspring. J Assist Reprod Genet.

[B11] Osada H, Aono F, Kuwayama M, Morita H, Teramoto S, Kato O (2003). Clinical efficiency of vitrification on blastocysts transfer cycles. Fertil Steril.

[B12] Stehlik E, Stehlik J, Katayama KP, Kuwayama M, Jambor V, Brohammer R, Kato O (2005). Vitrification demonstrates significant improvement versus slow freezing of human blastocysts. Reprod Biomed Online.

[B13] Liebermann J, Tucker MJ (2006). Comparison of vitrification and conventional cryopreservation of day 5 and day 6 blastocysts during clinical application. Fertil Steril.

[B14] Liebermann J, Nawroth F, Isachenko V, Isachenko E, Rahimi G, Tucker MJ (2002). Potential importance of vitrification in reproductive medicine. Biol Reprod.

[B15] Vajta G, Holm P, Kuwayama M, Booth PJ, Jacobsen H, Greve T, Callesen H (1998). Open Pulled Straw (OPS) vitrification: a new way to reduce cryoinjuries of bovine ova and embryos. Mol Reprod Dev.

[B16] Cremades N, Sousa M, Silva J, Viana P, Sousa S, Oliveira C, Teixeira da Silva J, Barros A (2004). Experimental vitrification of human compacted morulae and early blastocysts using fine diameter plastic micropipettes. Hum Reprod.

[B17] Kuwayama M, Vajta G, Kato O, Leibo SP (2005). Highly efficient vitrification method for cryopreservation of human oocytes. Reprod Biomed Online.

[B18] Kuwayama M, Vajta G, Ieda S, Kato O (2005). Comparison of open and closed methods for vitrification of human embryos and the elimination of potential contamination. Reprod Biomed Online.

[B19] Takahashi K, Mukaida T, Goto T, Oka C (2005). Perinatal outcome of blastocyst transfer with vitrification using cryoloop: a 4-year follow-up study. Fertil Steril.

[B20] Liebermann J (2009). Vitrification of human blastocysts: An update. Reprod Biomed Online.

[B21] Cho HJ, Son WY, Yoon SH, Lee SW, Lim JH (2002). An improved protocol for dilution of cryoprotectants from vitrified human blastocysts. Hum Reprod.

[B22] Fisch JD, Rodriguez H, Ross R, Overby G, Sher G (2001). The Graduated Embryo Score (GES) predicts blastocyst formation and pregnancy rate from cleavage-stage embryos. Hum Reprod.

[B23] Plachot M, Belaisch-Allart J, Mayenga JM, Chouraqui A, Serkine AM, Tesquier L (2000). Blastocyst stage transfer: the real benefits compared with early embryo transfer. Hum Reprod.

[B24] Rienzi L, Ubaldi F, Iacobelli M, Ferrero S, Minasi MG, Martinez F, Tesarik J, Greco E (2002). Day 3 embryo transfer with combined evaluation at the pronuclear and cleavage stages compares favourably with day 5 blastocyst transfer. Hum Reprod.

[B25] Shapiro BS, Harris DC, Richter KS (2000). Predictive value of 72-hour blastomere cell number on blastocyst development and success of subsequent transfer based on the degree of blastocyst development. Fertil Steril.

[B26] Bogliolo L, Ariu F, Fois S, Rosati I, Zedda MT, Leoni G, Succu S, Pau S, Ledda S (2007). Morphological and biochemical analysis of immature ovine oocytes vitrified with or without cumulus cells. Theriogenology.

[B27] Rho GJ, Kim S, Yoo JG, Balasubramanian S, Lee HJ, Choe SY (2002). Microtubulin configuration and mitochondrial distribution after ultra-rapid cooling of bovine oocytes. Mol Reprod Dev.

[B28] Noyes N, Porcu E, Borini A (2009). Over 900 oocyte cryopreservation babies born with no apparent increase in congenital anomalies. Reprod Biomed Online.

[B29] Mukaida T, Takahashi K, Goto T, Oka C (2008). Perinatal outcome of vitrified human blastocysts in 7 year experience (2670 attempted cycles). Human Reproduction.

[B30] Vanderzwalmen P, Bertin G, Debauche C, Standaert V, Bollen N, van Roosendaal E, Vandervorst M, Schoysman R, Zech N (2003). Vitrification of human blastocysts with the Hemi-Straw carrier: application of assisted hatching after thawing. Hum Reprod.

[B31] Barrenetxea G, Lopez de Larruzea A, Ganzabal T, Jimenez R, Carbonero K, Mandiola M (2005). Blastocyst culture after repeated failure of cleavage-stage embryo transfers: a comparison of day 5 and day 6 transfers. Fertil Steril.

[B32] Shapiro BS, Richter KS, Harris DC, Daneshmand ST (2001). A comparison of day 5 and day 6 blastocyst transfers. Fertil Steril.

[B33] Richter KS, Shipley SK, McVearry I, Tucker MJ, Widra EA (2006). Cryopreserved embryo transfers suggest that endometrial receptivity may contribute to reduced success rates of later developing embryos. Fertil Steril.

[B34] Van Voorhis BJ, Dokras A (2008). Delayed blastocyst transfer: is the window shutting?. Fertil Steril.

[B35] Veeck LL, Bodine R, Clarke RN, Berrios R, Libraro J, Moschini RM, Zaninovic N, Rosenwaks Z (2004). High pregnancy rates can be achieved after freezing and thawing human blastocysts. Fertil Steril.

[B36] Kader A, Agarwal A, Abdelrazik H, Sharma RK, Ahmady A, Falcone T (2009). Evaluation of post-thaw DNA integrity of mouse blastocysts after ultrarapid and slow freezing. Fertil Steril.

[B37] Vanderzwalmen P, Bertin G, Debauche C, Standaert V, van Roosendaal E, Vandervorst M, Bollen N, Zech H, Mukaida T, Takahashi K, Schoysman R (2002). Births after vitrification at morula and blastocyst stages: effect of artificial reduction of the blastocoelic cavity before vitrification. Hum Reprod.

[B38] Leoni GG, Berlinguer F, Succu S, Bebbere D, Mossa F, Madeddu M, Ledda S, Bogliolo L, Naitana S (2008). A new selection criterion to assess good quality ovine blastocysts after vitrification and to predict their transfer into recipients. Mol Reprod Dev.

[B39] Pribenszky C, Cseh S, Abonyi-Toth Z, Solti L (2003). Survival of rapidly frozen hatched mouse blastocysts. Zygote.

[B40] Zech NH, Lejeune B, Zech H, Vanderzwalmen P (2005). Vitrification of hatching and hatched human blastocysts: effect of an opening in the zona pellucida before vitrification. Reprod Biomed Online.

[B41] Mukaida T, Oka C, Goto T, Takahashi K (2006). Artificial shrinkage of blastocoeles using either a micro-needle or a laser pulse prior to the cooling steps of vitrification improves survival rate and pregnancy outcome of vitrified human blastocysts. Hum Reprod.

[B42] Chen SU, Lee TH, Lien YR, Tsai YY, Chang LJ, Yang YS (2005). Microsuction of blastocoelic fluid before vitrification increased survival and pregnancy of mouse expanded blastocysts, but pretreatment with the cytoskeletal stabilizer did not increase blastocyst survival. Fertil Steril.

[B43] Son WY, Yoon SH, Yoon HJ, Lee SM, Lim JH (2003). Pregnancy outcome following transfer of human blastocysts vitrified on electron microscopy grids after induced collapse of the blastocoele. Hum Reprod.

[B44] Hiraoka K, Kinutani M, Kinutani K (2004). Blastocoele collapse by micropipetting prior to vitrification gives excellent survival and pregnancy outcomes for human day 5 and 6 expanded blastocysts. Hum Reprod.

[B45] Kader A, Sharma RK, Falcone T, Agarwal A (2009). Mouse blastocyst previtrification interventions and DNA integrity. Fertil Steril.

[B46] Li R, Trounson A (1991). Rapid freezing of the mouse blastocyst: effects of cryoprotectants and of time and temperature of exposure to cryoprotectant before direct plunging into liquid nitrogen. Reprod Fertil Dev.

[B47] Dumoulin JC, Bergers-Janssen JM, Pieters MH, Enginsu ME, Geraedts JP, Evers JL (1994). The protective effects of polymers in the cryopreservation of human and mouse zonae pellucidae and embryos. Fertil Steril.

[B48] Zhu SE, Zeng SM, Yu WL, Li SJ, Zhang ZC, Chen YF (2001). Vitrification of in vivo and in vitro produced ovine blastocysts. Anim Biotechnol.

[B49] Ohboshi S, Fujihara N, Yoshida T, Tomogane H (1997). Usefulness of polyethylene glycol for cryopreservation by vitrification of in vitro-derived bovine blastocysts. Anim Reprod Sci.

[B50] Mahmoudzadeh AR, Van Soom A, Bols P, Ysebaert MT, de Kruif A (1995). Optimization of a simple vitrification procedure for bovine embryos produced in vitro: effect of developmental stage, two-step addition of cryoprotectant and sucrose dilution on embryonic survival. J Reprod Fertil.

[B51] Kaidi S, Van Langendonckt A, Massip A, Dessy F, Donnay I (1999). Cellular alteration after dilution of cryoprotective solutions used for the vitrification of in vitro-produced bovine embryos. Theriogenology.

[B52] Cseh S, Horlacher W, Brem G, Corselli J, Seregi J, Solti L, Bailey L (1999). Vitrification of mouse embryos in two cryoprotectant solutions. Theriogenology.

[B53] Mayer E (1988). Hyperquenching of water and dilute aqueous-solutions into their glassy states- An approach to cryofixation. Cryo-Lett.

[B54] Yavin S, Arav A (2007). Measurement of essential physical properties of vitrification solutions. Theriogenology.

[B55] Arav A, Zeron Y (1997). Vitrification of bovine oocytes using modified minimum drop size technique (MDS) is effected by the composition and the concentration of the vitrification solution and by the cooling conditions. Theriogenology.

[B56] Bielanski A, Bergeron H, Lau PC, Devenish J (2003). Microbial contamination of embryos and semen during long term banking in liquid nitrogen. Cryobiology.

[B57] Fountain D, Ralston M, Higgins N, Gorlin JB, Uhl L, Wheeler C, Antin JH, Churchill WH, Benjamin RJ (1997). Liquid nitrogen freezers: a potential source of microbial contamination of hematopoietic stem cell components. Transfusion.

[B58] Isachenko V, Katkov II, Yakovenko S, Lulat AG, Ulug M, Arvas A, Isachenko E (2007). Vitrification of human laser treated blastocysts within cut standard straws (CSS): novel aseptic packaging and reduced concentrations of cryoprotectants. Cryobiology.

[B59] Isachenko V, Alabart JL, Nawroth F, Isachenko E, Vajta G, Folch J (2001). The open pulled straw vitrification of ovine GV-oocytes: positive effect of rapid cooling or rapid thawing or both?. Cryo Letters.

[B60] Huang CC, Lee TH, Chen SU, Chen HH, Cheng TC, Liu CH, Yang YS, Lee MS (2005). Successful pregnancy following blastocyst cryopreservation using super-cooling ultra-rapid vitrification. Hum Reprod.

[B61] Santos RMd, Barreta MH, Frajblat M, Cucco DC, Mezzalira JC, Bunn S, Cruz FB, Vieira AD, Mezzalira A (2006). Vacuum-cooled liquid nitrogen increases the developmental ability of vitrified-warmed bovine oocytes. Ciência Rural.

[B62] Arav A, Zeron Y, Ocherenty A (2000). A new device and method for vitrification increases the cooling rates and allows successful cryopreservation of bovine oocytes. Theriogenology.

[B63] Yavin S, Aroyo A, Roth Z, Arav A (2009). Embryo cryopreservation in the presence of low concentration of vitrification solution with sealed pulled straws in liquid nitrogen slush. Hum Reprod.

[B64] Kaarniranta K, Elo M, Sironen R, Lammi MJ, Goldring MB, Eriksson JE, Sistonen L, Helminen HJ (1998). Hsp70 accumulation in chondrocytic cells exposed to high continuous hydrostatic pressure coincides with mRNA stabilization rather than transcriptional activation. Proceedings of the National Academy of Sciences of the United States of America.

[B65] Wemekamp-Kamphuis HH, Karatzas AK, Wouters JA, Abee T (2002). Enhanced Levels of Cold Shock Proteins in Listeria monocytogenes LO28 upon Exposure to Low Temperature and High Hydrostatic Pressure. Appl Environ Microbiol.

[B66] Kuo Y-H, Pribenszky C, Huang S-Y (2008). Higher litter size is achieved by the insemination of high hydrostatic pressure-treated frozen-thawed boar semen. Theriogenology.

[B67] Pribenszky C, Du Y, Molnár M, Harnos A, Vajta G (2008). Increased stress tolerance of matured pig oocytes after high hydrostatic pressure treatment. Animal Reproduction Science.

[B68] Pribenszky C, Molnar M, Cseh S, Solti L (2005). Improving post-thaw survival of cryopreserved mouse blastocysts by hydrostatic pressure challenge. Anim Reprod Sci.

[B69] Du Y, Pribenszky CS, Molnar M, Zhang X, Yang H, Kuwayama M, Pedersen AM, Villemoes K, Bolund L, Vajta G (2008). High hydrostatic pressure: a new way to improve in vitro developmental competence of porcine matured oocytes after vitrification. Reproduction.

[B70] Son WY, Tan SL (2009). Comparison between slow freezing and vitrification for human embryos. Expert Rev Med Devices.

[B71] Youssry M, Ozmen B, Zohni K, Diedrich K, Al-Hasani S (2008). Current aspects of blastocyst cryopreservation. Reprod Biomed Online.

